# Ongoing Treatment with a Spore-Based Probiotic Containing Five Strains of *Bacillus* Improves Outcomes of Mild COVID-19

**DOI:** 10.3390/nu15030488

**Published:** 2023-01-17

**Authors:** Adrian Catinean, Anamaria Sida, Celina Silvestru, Gheorghe G. Balan

**Affiliations:** 1 Department of Internal Medicine, Iuliu Hatieganu University of Medicine and Pharmacy, 400006 Cluj-Napoca, Romania; 2 Department of Gastroenterology, Grigore T. Popa University of Medicine and Pharmacy, 700115 Iasi, Romania

**Keywords:** *Bacillus*, COVID-19, probiotics, SARS-CoV-2, time to symptom resolution, gut microbiota, gut–lung axis, gastrointestinal COVID-19 symptoms, immunomodulation, fever in COVID-19 infection

## Abstract

Spore-based *Bacillus* probiotic treatment improves intestinal health. The intestinal microbiota influences both the innate and adaptive immune responses. As such, the influence of ongoing spore-based probiotic treatment (five probiotic strains of *Bacillus*) on the clinical outcomes of mild COVID-19 was evaluated in this retrospective, observational study. Demographics, medical history, probiotic use, and COVID-19 symptom information were collected. The study included 120 patients with a PCR-confirmed SARS-CoV-2 infection and mild COVID-19 symptoms. The probiotic group (*n* = 60) comprised patients with ongoing probiotic treatment (≥1 month); the control group comprised patients not taking probiotics (*n* = 60). The primary outcome was time to symptom resolution; secondary outcomes included time to fever resolution and presence of digestive symptoms. The probiotic group had a significantly shorter time to symptom resolution (mean (95% confidence interval) days: control group, 8.48 (6.56, 10.05); probiotic group, 6.63 (5.56; 6.63); *p* = 0.003) and resolution of fever (control group, 2.67 (1.58, 3.61); probiotic group, 1.48 (1.21, 2.03); *p* < 0.001). More patients in the probiotic group (*n* = 53) than in the control group (*n* = 34) did not have digestive symptoms (*p* < 0.001). Among adults with mild COVID-19, participants receiving ongoing probiotic treatment had a shorter clinical course, and fewer had digestive symptoms compared with those not taking probiotics.

## 1. Introduction

The COVID-19 pandemic has changed paradigms in both medicine and society across the globe. The World Health Organization (WHO) reports that, to date, severe acute respiratory syndrome coronavirus 2 (SARS-CoV-2), the beta-coronavirus virus that causes COVID-19 [1], has infected over 661 million people worldwide and there have been nearly 7 million deaths [2]. Considering the challenges to biological containment of SARS-CoV-2 in the population, it has been necessary to develop research strategies to identify targets that may be useful to prevent the spread of SARS-CoV-2 and to reduce the severity of COVID-19 symptoms. Such strategies have been fueled by evidence obtained through COVID-associated research programs.

In the field of gastrointestinal (GI) diseases, special attention has been directed to-wards elucidating how the human microbiota is able to modulate systemic immune responses [3,4,5,6]. Given the influence of the gut microbiome on systemic immune responses, researchers have begun to evaluate whether the gut microbiome plays a role in COVID-19 outcomes, and whether disease outcomes may be improved through manipulation of the gut microbiome. An increasing number of ongoing studies are focused on finding associations between the composition and activity of the gut microbiome and COVID-19 disease outcomes. To identify new ways to prevent or treat SARS-CoV-2 infection, it is important to understand how the virus infects cells and what mechanisms are involved in disease progression. Given the association between higher pro-inflammatory cytokine levels and admission to intensive care units for COVID-19 symptoms [7], one possible mechanism to prevent severe disease is through the suppression of the immune response triggered by SARS-CoV-2 infection. Indeed, the effects of treatments that suppress cytokine storm are being tested in patients with COVID-19. Other potential strategies that have been or are currently being evaluated include the prevention of viral entry and/or replication [8]. Such studies will provide insight into the mechanisms by which the virus infects cells and causes disease progression, potentially providing new strategies for the prevention of infection and/or treatment of disease.

The severity of COVID-19 ranges from mild to severe and life-threatening [1]. Infected patients can be asymptomatic or experience a range of symptoms from mild, non-specific flu-like symptoms (e.g., fever, cough, or sore throat) to severe illness or even death [1]. The symptoms of COVID-19 disease affect not only the pulmonary system, but also the gastrointestinal, neurological, and cardiovascular systems. Additionally, patients may have atypical presentations such as cutaneous, ophthalmic, and gustarory manifestations [9]. A study of patients 6 months after their COVID-19 diagnosis found that a gastrointestinal symptom was considered as the most bothersome symptom by 11% of all patients in the study [10]. A study in China reported that approximately one in two patients with COVID-19 experienced at least one digestive manifestation, including heartburn, constipation, diarrhea, and abdominal pain [11].

The human intestinal microbiota is a complex system within the body that plays several diverse and important roles and has anti-inflammatory, immunomodulatory, and antioxidant properties [1]. Compared to the intestinal microbiota, the number of microbes in the lung is very small. However, there is a two-way communication system between the intestine and the lung called the gut-lung axis which plays a major role in maintaining immune homeostasis. Thus, intestinal inflammation may be correlated with inflammation of the lungs and the occurrence of dysbiosis in the intestine could be associated with severe respiratory disorders [12]. Long-lasting COVID-19 gastrointestinal symptoms, such as diarrhea, have been associated with reduced gut microbiota richness and diversity, immunological dysregulation, and slower SARS-CoV-2 clearance. Therefore, the gut-lung axis—a bidirectional relationship between the respiratory mucosa and the gut microbiota—is thought to play a role in either healthy or pathological immunological reactions to SARS-CoV-2 [13]. Changes in the intestinal microbiota that have a beneficial effect on human health can be achieved by administering probiotics, which are defined by the WHO as ”live microorganisms which when administered in adequate amounts confer a health benefit to the host” [14]. Probiotics exert their beneficial effects through various mechanisms, including altering the immunological response of the host, lessening pathogenic organism colonization and invasion, and lowering pH. Traditionally, the treatment of acute diarrhea has provided the most convincing evidence of the clinical benefit of probiotics. Benefits of probiotics linked to one strain or species might not always apply to others. Probiotics are generally considered safe and well tolerated, with bloating and flatulence being the most common side effects [15].

Despite all the recent research in the field, there are still limited data in the literature describing the link between intestinal microbes and the susceptibility to SARS-CoV-2 infection or severity of COVID-19 disease. To help address this paucity of data, we investigated whether a spore-based probiotic (Megasporebiotic™) containing five strains of *Bacillus* spores (*Bacillus indicus* [HU36], *Bacillus subtilis* [HU58], *Bacillus coagulans* [SC208], *Bacillus licheniformis* [SL307], and *Bacillus clausii* [SC109]) had an effect on the duration or severity of symptoms in adults with mild COVID-19.

Unquestionably, the global public health crisis caused by the COVID-19 pandemic has escalated. Public health concerns associated with the pandemic are linked to the virus’ ability to be easily transmitted from person to person and the ability of the virus to quickly mutate, resulting in the formation of novel viral strains. This potential for change provides a challenge to finding new, efficient ways to treat COVID-19. Given that probiotics have been considered for their therapeutic potential in multiple illnesses, the aim of this work was to demonstrate whether patients who consistently self-administered a *Bacillus*-based probiotic for at least one month prior to being diagnosed with COVID-19 had any difference in symptom severity or overall symptom resolution compared with patients who were not taking probiotics.

## 2. Participants and Methods

### 2.1. Study Design

This observational, retrospective study was conducted between 15 September 2020 and 15 February 2021 at Diasan Medical Centre in Cluj-Napoca, Romania. The study protocol was approved by the Ethics Committee of the Universitatea de Medicină și Farmacie Iuliu Hatieganu (registry number, 278/30.06.2021). Participation in this study was voluntary; completion of the questionnaire provided consent to participate.

This study was initiated based on the clinical observation that individuals receiving the *Bacillus*-spore-based probiotic had milder symptoms and a shorter course of illness when infected with SARS-CoV-2. We recruited individuals receiving ongoing probiotic treatment (two capsules containing 4 billion colony-forming units per capsule, taken once daily with the mid-day meal), who had contracted SARS-CoV-2 and developed symptoms of COVID-19, to participate in this study by filling out a questionnaire at their visit to our clinic following their positive COVID-19 diagnosis. These participants were matched with controls who were not receiving ongoing probiotic treatment, who had contracted SARS-CoV-2 and developed mild COVID-19 symptoms. Controls were selected by applying specific filters (mild cases, age, and BMI to match the probiotic group) to the electronic database of the Arad, Maramureș and Cluj County Health Departments, which tracked patients with COVID-19. Following identification of appropriate control participants, those identified were contacted and asked to complete the study questionnaire. Participants were permitted to take other medications during the study; treatment for COVID-19 was provided to participants according to the COVID-19 treatment protocols at each participating institution and at the discretion of the treating physician. 

The questionnaire included 21 questions regarding sex, age, weight, height, SARS-CoV-2 PCR test, history of chronic diseases, history of treatment for chronic diseases, duration of probiotic treatment, medications taken outside the protocol prescribed by the attending physician, duration, severity, and presence of digestive symptoms experienced during COVID-19 illness (Text S1). Following completion of the questionnaire, personal information such as full name, address, and phone number were redacted to ensure confidentiality.

### 2.2. Participants

Participants between 18 and 65 years of age, who had a positive SARS-CoV-19 PCR test using a sample collected from nasal or pharyngeal secretions within the 7 days prior to completing the questionnaire, were included.

Participants were excluded if they had a New York Heart Association Classification of Heart Failure greater than 2, a diagnosis of severe chronic obstructive pulmonary disease, a neoplastic disease and were undergoing chemo-radiotherapy (except for those individuals in documented, clinical, serological, and imaging remission), type 1 diabetes, or were pregnant.

Participants were divided into two groups according to whether they had been treated with probiotic for at least one month (probiotic group) or had not received any probiotic treatment (control group)

### 2.3. Outcomes

The primary outcome was time to resolution of all symptoms. Secondary outcomes were time to fever resolution, maximum fever, and the presence of digestive symptoms.

### 2.4. Statistical Analysis

Given that this was a retrospective study, sample size was determined by the number of available people who were receiving ongoing spore-based probiotic treatment and contracted SARS-CoV-2 with mild symptoms of COVID-19. We also considered the sample sizes of previously published reports of patients receiving nutritional support or probiotics who received a COVID-19 or influenza vaccination [16] or were diagnosed with COVID-19 [17,18].

Data analyses were conducted using Pearson’s chi-squared test, the independent samples Student’s *t*-test, the Mann–Whitney U test, Fisher’s exact test, and the log-rank survival test. All analyses were performed using the Statistical Package for Social Sciences software (SPSS Inc., Chicago, IL, USA), version 20.0.0.

## 3. Results

### 3.1. Participants

**Figure 1:**
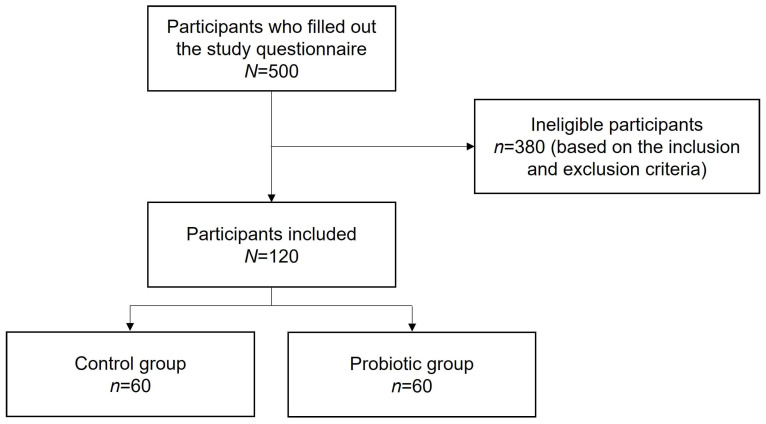
Participant disposition.

During the study period, 500 individuals who had recovered from COVID-19 were asked to fill out the questionnaire; 120 participants, who met the inclusion criteria and did not meet the exclusion criteria, were, thus, eligible to participate in the study. Sixty participants were included in the control group, and sixty were in the probiotic group (Figure 1).

Participant demographic characteristics are shown in Table 1. The median age of participants in both groups was 41 years, and age was normally distributed in both groups. The mean body mass index (BMI) in the control and probiotic groups was 26.91 and 25.91, respectively. The between-group differences for age and BMI were not significant.

Participants were treated with vitamin C, vitamin D, zinc, quercetin supplements, acetaminophen, non-steroidal anti-inflammatory drugs, and antibiotics. The main antibiotic used was azithromycin, which was combined with third-generation cephalosporin in some participants. In total, 37 participants in the control group and 13 in the probiotic group received antibiotic treatment during the study.

### 3.2. Primary Outcome

**Figure 2:**
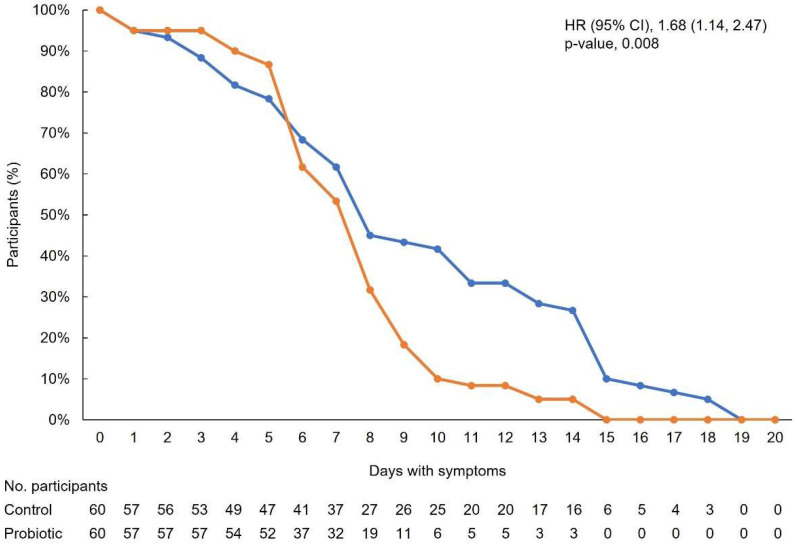
Time to resolution of all COVID-19 symptoms. The orange line represents participants in the probiotic group, and the blue line represents participants in the control group. CI, confidence interval; HR, hazard ratio.

The primary outcome of time to resolution of symptoms was significantly shorter for participants in the probiotic group (mean (SD) 6.63 (2.92) days) versus the control group (8.48 (5.03) days) (log-rank test; *p* = 0.003) (Figure 2). The HR (95% CI) was 1.68 (1.14, 2.47) (*p* = 0.008), indicating that participants in the probiotic group were 1.68 times more likely to have a faster symptom resolution compared with controls.

### 3.3. Secondary Outcomes

**Figure 3:**
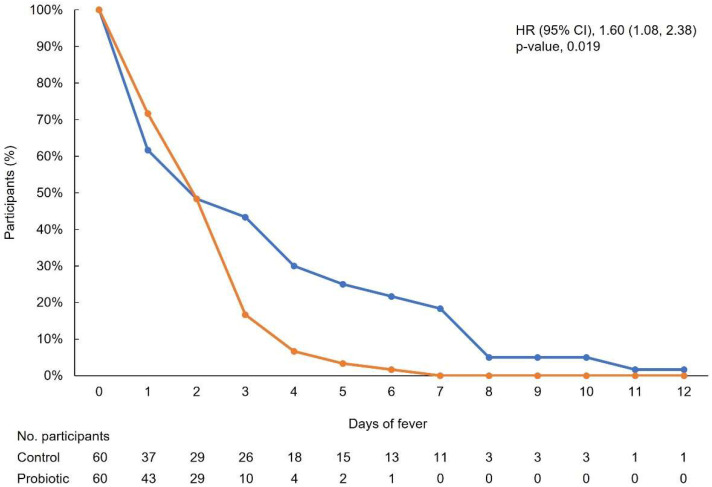
Kaplan–Meier curve showing time to resolution of fever. The orange line represents participants in the probiotic group, and the blue line represents participants in the control group. CI, confidence interval; HR, hazard ratio.

Fever was resolved significantly faster in participants in the probiotic group versus the control group (log-rank test; *p* = 0.004) (Figure 3). The HR (95% CI) was 1.60 (1.08, 2.38) (*p* = 0.019), indicating that participants in the probiotic group were 1.60 times more likely to have their fever resolved sooner versus those in the control group.

**Figure 4:**
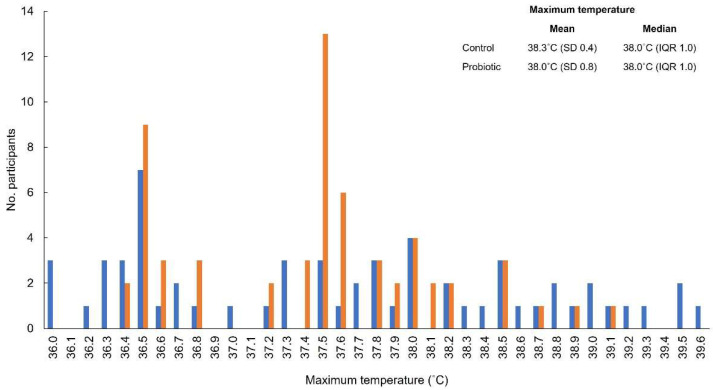
Distribution of maximum fever for individual participants. The orange bars represent participants in the probiotic group, and the blue bars represent participants in the control group. IQR, interquartile range; SD, standard deviation.

The distribution of maximum fever for individual participants is shown in Figure 4. The mean (standard deviation) maximum temperature was similar between participants in the probiotic and control groups (probiotic group, 38.0 °C (0.434)); control group, 38.3 °C (0.777)). The median (interquartile range) for both groups was 38.0 °C (1.0).

Digestive symptoms were present in a significantly lower proportion of participants in the probiotic group versus the control group (11.7% (*n* = 7/60) versus 43.3% (*n* = 26); *p* < 0.001).

No data were missing for any of the primary or secondary outcome measures.

## 4. Discussion

In this retrospective study of symptomatic adults with COVID-19, participants who were treated with a spore-based probiotic for ≥1 month prior to infection had a significantly faster time to symptom resolution.

The five strains of *Bacillus* included in the probiotic support a healthy gut microbiome, which in turn bolsters healthy digestion and immune function [19,20,21]. Specifically, unlike non-spore-based probiotic products, these *Bacillus* spores survive gastrointestinal passage and transform into the active, vegetative form, temporarily colonizing the gut upon reaching the large intestine [22]. The *Bacillus* spores are able to modulate inflammation and boost short-chain fatty acid (SCFA) production through a reconditioning of the gut microbiota [19,20,21,23]. According to one study, a *B. coagulans*-based probiotic enhanced quality of life and reduced gastrointestinal discomfort in people with postprandial intestinal gas-related symptoms [24]. SARS-CoV-2 gains entry into cells via binding of the viral spike protein to the angiotensin-converting enzyme 2 (ACE-2) receptor on host cells; thus, higher ACE-2 expression levels may increase susceptibility to COVID-19 [25]. ACE-2 is expressed on the surface of lung alveolar epithelial cells and enterocytes of the small intestine, as well as arterial and venous endothelial cells, and smooth muscle cells, with the most abundant expression being observed on the epithelia of the lung and small intestine [26]. A recent study found that nattokinase, a serine protease produced by *B. subtilis var. natto*, is able to degrade the SARS-CoV-2 spike protein, potentially limiting the viral replication and cell entry [27]; this finding may explain the mechanism behind the *in vitro* observation that SARS-CoV-2 infection was inhibited by natto extract [28].

It has been well-documented that SARS-CoV-2 affects the lower respiratory tract, where it can manifest as pneumonia [29]. In addition to this, it is known that digestive symptoms are also somewhat common in patients infected with SARS-CoV-2 [30]. COVID-19 can progress rapidly in some patients, resulting in acute respiratory distress syndrome, multiorgan failure, and death [29]. A recent study found that SARS-CoV-2 can lead to severe outcomes through endothelial cell activation. Specifically, the SARS-CoV-2 nucleocapsid protein was shown to activate human endothelial cells via the Toll-like receptor 2/nuclear factor kappa-light-chain-enhancer of activated B cells pathways. Nucleocapsid protein significantly increases the expression of the endothelial cell activation markers intracellular adhesion molecule 1 (ICAM-1) and vascular cell adhesion molecule-1 (VCAM-1) [31]. Interestingly, a recent study in rats reported that the spore-based probiotic, Megasporebiotic™, lowered serum ICAM-1 levels and, to a lesser extent, lowered serum VCAM-1 levels in the context of inflammatory bowel disease (IBD) [32]. These findings are supported by the present study, which found that the overall duration of symptoms was shorter.

Fever is a common symptom in many viral infections [33] and is the earliest and most common symptom to appear for patients with COVID-19; however, the absence of fever does not exclude infection [29]. Fever is a rise in body temperature above the normal range that occurs as a result of elevation of the hypothalamic set point [33]. It is widely understood in clinical practice that viruses can cause fever by direct recruitment of inflammatory cells [34], antibody production [33], stimulating increased type 1 interferon (IFN-I) activity [35], and virus-medicated necrosis [33,36]. In our study, fever was defined as a temperature of ≥37.5 °C, with duration calculated from the date of onset until the date the temperature was reduced to <37.5 °C for >24 h. Both groups similarly experienced fever (probiotic group, *n* = 43; control group, *n* = 37; *p* = 0.25); however, the number of days with fever was significantly lower in the probiotic group versus the control (2.07 versus 4.32; *p* < 0.001), as was the maximum recorded temperature (*p* = 0.003).

Fever is an important symptom because it can result in a “cytokine storm”, in which IL-1, 2, 6, 7, 8, 10, 12, 17, and18; tumor necrosis factor α (TNF-α); IFN-y; granulocyte colony-stimulating factor; granulocyte-macrophage colony-stimulating factor; and monocyte chemoattractant protein-1 are released [37]. IL-6 is a major pyrogenic agent that participates in raising body temperature through autonomous thermoregulatory mechanisms [38]. Probiotics containing strains of *Lactobacillus* or *Biffidobacterium* have been the focus of most studies assessing the therapeutic effects of probiotics in patients with SARS-CoV-2 infection [1,39,40,41]. Prior to the present study, the role of spore-based probiotics in COVID-19 had not been studied. However, the ability of this spore-based probiotic to modulate inflammation has been demonstrated in a study of rats with induced ulcerative colitis. Megasporebiotic™ decreased the levels of IL-6 and TNF-α in this model [32]. Thus, we speculate that the favorable effect of this spore-based probiotic on fever was due to its effect on proinflammatory markers, especially IL-6.

Around 15% of patients with COVID-19 have gastrointestinal symptoms, with the three most common of these being nausea or vomiting, loss of appetite, and diarrhea [30]. It is known that the microbiota is affected by SARS-CoV-2 [42]. Compared with the healthy controls, patients with COVID-19 had significantly reduced bacterial diversity and a considerably higher relative abundance of opportunistic pathogens [43,44]. Of note, none of the participants in either group were being treated with steroids or other therapies intended to reduce the intestinal symptoms associated with IBS and small intestinal bacterial overgrowth (e.g., mesalazines, 5-aminosalicylic acids, and biologics) at the time of the study. Both the gastrointestinal and airway microbiotas of healthy people include a range of bacteria, including members of the Bacteroidetes and Firmicutes phylums. A dysbiosis of both the gastrointestinal and airway microbiota was reported during respiratory disease, commonly presenting as an outgrowth of the Proteobacteria and Firmicutes phylums [45]. However, it turns out that the intestinal microbiota also influences the pulmonary microbial composition and immune responses, by both the direct seeding of the respiratory tract with bacteria and the distribution of bacterial metabolites, such as SCFAs, which provide fuel for colonocytes and/or act directly as immunomodulatory molecules [46,47]. Likewise, the probiotic strains in the spore-based probiotic affect the structure and function of the gut microbiota through increased SCFA (acetate, propionate, and butyrate) production by *Firmicutes* [19,23]. Additionally, SARS-CoV-2-infected patients have an altered stool microbiome composition compared to controls [43]. In hospitalized patients with COVID-19, stool levels of several commensal gut bacteria (including *Bacteroides dorei* and *Bacteroides ovatus*) were found to correlate inversely with fecal SARS-CoV-2 viral load. This is of interest, given that such bacteria have been shown to downregulate the expression of angiotensin-converting enzyme 2 (ACE2) in the murine gut [48], which is utilized by SARS-CoV-2 to gain entry to the host. Thus, digestive symptoms that occur in SARS-CoV-2 infection may be caused by direct and/or immune-mediated viral invasion. The beneficial effects of the spore-based probiotic in SARS-CoV-2 infection can be attributed to a variety of characteristics for the *Bacillus* strains included in the formulation, and the effects are assumed to be both indirect (e.g., through modulation of the gut microbiome composition and activity and immunomodulation) and direct. An example of a possible direct effect of *B. subtilis* is the production of a biosurfactant that is able to render enveloped viruses inactive [49].

A key feature of COVID-19 is the overwhelming inflammation observed in some patients, especially those who develop severe illness [50]. The human body responds to SARS-CoV-2 infection via both the innate and adaptive immune responses. Innate immune cells release cytokines IFN, though the levels of IFN released in response to SARS-CoV-2 are low relative to the response to other viral infections [51]. These inflammatory signals, in turn, start the adaptive immune response. Levels of IL-6, TNFα, IL-10, and other cytokines are significantly increased with infection [52]; elevation of IL-6 and IL-10 is a reliable indicator of a more severe disease [53]. A delayed IFN response by the innate immune system may account for the uninterrupted initial viral replication and delayed priming of the adaptive immune response observed in some patients. These patients are at a greater risk for a more severe disease, as a higher viral load is associated with a risk of intubation and fatality [54]. An increased viral load is also associated with an increase in IL-6 release [50,51]. The non-structural proteins of SARS-CoV-2 can induce the expression of several cytokines via activation of the NFκB pathway [7]. Moreover, bacterial lipopolysaccharide, pro-inflammatory cytokines such as TNF-α and IL-1, and viral infections may all activate NFκB, and extensive mutational analyses have revealed the NFκB binding region as being critical for IL-6 gene activation [55]. A 2017 study by McFarlin et al. found that *Bacillus*-spore-based probiotic supplementation in participants who exhibited signs of post-prandial dietary endotoxemia resulted in a reduction in several biomarkers of inflammation (IL-12p70, IL-1β, IL-6, IL-8, and MCP-1) [21]. This is consistent with our findings, which showed that patients receiving a spore-based probiotic had a better clinical progression than those who did not, in terms of fever and total days with symptoms.

## 5. Conclusions

Due to the increased geographic spread of the SARS-CoV-2 virus over a short span of time, the virus is considered a global health hazard and has contributed to increased mortality rates, had notable effects on the economy, and exhausted medical resources around the world. Being a new disease, COVID-19 has presented itself to the medical community as a puzzling illness that has necessitated extensive study to uncover insights into the biology of the SARS-CoV-2 virus [56] Considering this, it has been extremely important to find safe, inexpensive, and readily available treatment strategies. While effective vaccines and targeted drugs have been and continue to be developed, alternative pathophysiology-based options to prevent and treat COVID-19 must be considered along with the limited evidence-based therapy currently available.

Due to the immunomodulatory properties of probiotics and prebiotics, peer-reviewed studies have led to the hypothesis that altering the gutlung microbiome could potentially be used as an adjuvant for the prevention and treatment of COVID-19, a conjecture that is supported by the present study. Our findings suggest that ongoing treatment with a spore-based probiotic contributes to a better clinical outcome for mild COVID-19, including fewer digestive symtoms, faster symtom resolution, and faster resolution of fever. 

Probiotics have many benefits, including balancing the human gut microbiota composition, enhancing the barrier function of the gut, and aiding the body's protective immune responses against pathogens. Together with current therapies, probiotic supplementation may play a role in COVID-19 disease mitigation and improved patient quality of life. However, the use of probiotic-based strategies for the treatment of COVID-19 in clinical settings is still an open research question. Therefore, to explore the benefits of probiotics in SARS-CoV-2 infection, multicenter clinical trials with large numbers of patients are need to confirm and expand the findings reported herein, and to explore whether other probiotic supplements may have similar effects.

Our findings suggest that consistently taking a spore-based probiotic before and during infection with SARS-CoV-2 positively impacts the course of COVID-19. Large studies are needed to better understand the gut-lung axis in relation to SARS-CoV-2 infection and COVID-19 disease progression.

## Figures and Tables

**Table 1:** Participant demographic characteristics.

	Control Group (*n* = 60)	Probiotic Group (*n* = 60)	*p*-Value
Age, years, mean (SD)	41.5 (13.6)	41.4 (12.1)	0.99 ^a^
BMI, mean (SD)	26.9 (4.8)	26.0 (5.5)	0.28 ^b^
Sex, male/female, *n* (%)	18/36 (33.3/66.7)	27/31 (46.6/53.5)	0.15 ^c^
SIBO, *n* (%)	0	10 (10.7)	<0.002 ^d^
AHT, *n* (%)	13 (21.7)	9 (15.0)	0.35 ^c^
Type 2 diabetes, *n* (%)	5 (8.3)	3 (5.0)	0.72 ^d^
IBS, *n* (%)	0	23 (38.33)	<0.001 ^d^
CD, *n* (%)	0	2 (3.33)	0.50 ^d^
UC, *n* (%)	0	4 (6.66)	0.12 ^d^

AHT, arterial hypertension; BMI, body mass index; CD, Crohn’s disease; IBS, irritable bowel syndrome; SIBO, small intestinal bacterial overgrowth; UC, ulcerative colitis. ^a^ Calculated using the independent samples *t*-test. ^b^ Calculated using the Mann–Whitney U test. ^c^ Calculated using Pearson’s chi-squared test. ^d^ Calculated using Fisher’s exact test.

## Data Availability

The data presented in this study are available on request from the corresponding author.

## Supplementary Materials

The following supporting information can be downloaded at: https://www.mdpi.com/article/10.3390/nu15030488/s1. Text S1: Questionnaire.
