# Supplementary material for: Ongoing Treatment with a Spore-Based Probiotic Containing Five Strains of Bacillus Improves Outcomes of Mild COVID-19
Source: Nutrients. 2023 Jan 17;15(3):488. doi: 10.3390/nu15030488 (PMC9920365; doi:10.3390/nu15030488)
Supplement: Supplementary file 1 [file nutrients-15-00488-s001.zip › nutrients-2134725-supplementary.pdf]

Questionnaire

Please take a few minutes to complete the following questionnaire:

1) First + Last name

2) Sex:

• Female

• Male

3) In the case of females, were you pregnant at the time of infection with the Sars-Cov2 virus?

5) Did you have your diagnosis of infection with the Sars-Cov2 virus confirmed by a positive PCR test?

• Yes

• Not

4) Age

6) What is your weight?

7) What is your height?

8) Did you have a fever during the Sars-Cov2 virus infection?

• Yes

• Not

9) If yes, how many days?

10) What is the maximum temperature you had during the Sars-Cov2 virus infection?

11) Did you have digestive symptoms during the Sars-Cov2 virus infection?

• Yes

• Not

12) If yes, how many days?

13) Do you have any chronic diseases? (for which you need to take chronic medication, or not necessarily)

• Yes

• Not

14) If so, what do you take?

15) Did you take Megasporebiotic treatment at least 1 month before the Sars-Cov2 infection?

• Yes

- Not

16) During the infection with the covid-19 virus, did you take antibiotics?

- Yes
- Not
